# Supplementary material for: End Sequence Analysis Toolkit (ESAT) expands the extractable information from single-cell RNA-seq data
Source: Genome Res. 2016 Oct;26(10):1397–410. doi: 10.1101/gr.207902.116 (PMC5052061; doi:10.1101/gr.207902.116)
Supplement: Supplemental Material [file supp_gr.207902.116_Supp_Materials.tar.gz › ESAT-esat_v0.1/doc/html/classbroad_1_1pda_1_1seq_1_1protection_1_1_two_sample_scan_skellam_peak_caller.html]

ESAT: broad.pda.seq.protection.TwoSampleScanSkellamPeakCaller Class Reference


ESAT


- Main Page
- Related Pages
- Packages
- Classes
- Files

- Class List
- Class Index
- Class Hierarchy
- Class Members

All Classes Namespaces Files Functions Variables Enumerator Pages

- broad
- pda
- seq
- protection
- TwoSampleScanSkellamPeakCaller

Public Member Functions |
Static Public Member Functions |
Static Public Attributes |
Protected Member Functions |
Static Protected Member Functions |
Protected Attributes |
Static Protected Attributes |
List of all members

broad.pda.seq.protection.TwoSampleScanSkellamPeakCaller Class Reference

Collaboration diagram for broad.pda.seq.protection.TwoSampleScanSkellamPeakCaller:

[legend]

|  |  |
| --- | --- |
| Public Member Functions | |
| Collection< Annotation > | filterWindows (Collection< Annotation > windows) throws IOException |
|  | |
| void | writeResults (Collection< Annotation > windows, String out) throws IOException |
|  | |
| void | writeResult (Collection< Annotation > windows, FileWriter writer) throws IOException |
|  | |

|  |  |
| --- | --- |
| Static Public Member Functions | |
| static double | getPoissonLambda (int regionLength, double d, int windowSize) |
|  | |

|  |  |
| --- | --- |
| Static Public Attributes | |
| static double | EXPRESSION\_PVALUE\_CUTOFF = 0.01 |
|  | |

|  |  |
| --- | --- |
| Protected Member Functions | |
| Annotation | trimPeak (Gene window) throws IOException |
|  | |

|  |  |
| --- | --- |
| Static Protected Member Functions | |
| static double | getSkellamPvalue (double backgroundLambda, double signalLambda, int backgroundCount, int signalCount) throws IOException |
|  | |

|  |  |
| --- | --- |
| Protected Attributes | |
| Map< String, Map< Gene, Double > > | backgroundScanPvalues |
|  | |
| ScanStatisticDataAlignmentModel | backgroundData |
|  | |
| String | backgroundName |
|  | |
| TranscriptomeSpaceAlignmentModel | signalData |
|  | |
| String | signalName |
|  | |
| Map< String, Collection< Gene > > | genes |
|  | |
| int | windowSize |
|  | |
| int | stepSize |
|  | |
| double | pValueCutoffSkellam |
|  | |
| double | pValueCutoffScan |
|  | |
| double | trimPeakByQuantile |
|  | |
| TranscriptomeSpace | transcriptomeSpace |
|  | |
| WindowProcessor< CountScore > | backgroundProcessor |
|  | |
| WindowProcessor  < ScanStatisticScore > | signalProcessor |
|  | |

|  |  |
| --- | --- |
| Static Protected Attributes | |
| static int | MIN\_WINDOW\_COUNT = 10 |
|  | |
| static Logger | logger = Logger.getLogger(TwoSampleScanSkellamPeakCaller.class.getName()) |
|  | |

## Detailed Description

Author
:   prussell

## Member Function Documentation

|  |  |  |  |  |  |
| --- | --- | --- | --- | --- | --- |
| Collection<Annotation> broad.pda.seq.protection.TwoSampleScanSkellamPeakCaller.filterWindows | ( | Collection< Annotation > | *windows* | ) | throws IOException |

|  |  |  |  |
| --- | --- | --- | --- |
| static double broad.pda.seq.protection.TwoSampleScanSkellamPeakCaller.getPoissonLambda | ( | int | *regionLength*, |
|  |  | double | *d*, |
|  |  | int | *windowSize* |
|  | ) |  |  |

static

Given a total number of reads, a region length, and a window size, get the parameter of the Poisson distribution modeling the number of reads in a window of this size

Parameters
:   |  |  |
    | --- | --- |
    | regionLength | The total region length |
    | d | The number of reads |
    | windowSize | The window size |

Returns
:   Lambda, the expected number of reads in the window under the null Poisson model

|  |  |  |  |
| --- | --- | --- | --- |
| static double broad.pda.seq.protection.TwoSampleScanSkellamPeakCaller.getSkellamPvalue | ( | double | *backgroundLambda*, |
|  |  | double | *signalLambda*, |
|  |  | int | *backgroundCount*, |
|  |  | int | *signalCount* |
|  | ) |  | throws IOException |

staticprotected

Compute Skellam P-value of read counts in a region given the two parameters

Parameters
:   |  |  |
    | --- | --- |
    | backgroundLambda | Poisson lambda for background sample |
    | signalLambda | Poisson lambda for signal sample |
    | backgroundCount | The count for background sample |
    | signalCount | The count for signal sample |

Returns
:   The probability under the null hypothesis of observing a greater difference

Exceptions
:   |  |  |
    | --- | --- |
    | IOException |  |

|  |  |  |  |  |  |
| --- | --- | --- | --- | --- | --- |
| Annotation broad.pda.seq.protection.TwoSampleScanSkellamPeakCaller.trimPeak | ( | Gene | *window* | ) | throws IOException |

protected

Trim the peak to max contiguous subsequence of counts above a certain quantile

Parameters
:   |  |  |
    | --- | --- |
    | window | The peak region |

Returns
:   The trimmed peak

Exceptions
:   |  |  |
    | --- | --- |
    | IOException |  |

|  |  |  |  |
| --- | --- | --- | --- |
| void broad.pda.seq.protection.TwoSampleScanSkellamPeakCaller.writeResult | ( | Collection< Annotation > | *windows*, |
|  |  | FileWriter | *writer* |
|  | ) |  | throws IOException |

|  |  |  |  |
| --- | --- | --- | --- |
| void broad.pda.seq.protection.TwoSampleScanSkellamPeakCaller.writeResults | ( | Collection< Annotation > | *windows*, |
|  |  | String | *out* |
|  | ) |  | throws IOException |

## Member Data Documentation

ScanStatisticDataAlignmentModel broad.pda.seq.protection.TwoSampleScanSkellamPeakCaller.backgroundData

protected

String broad.pda.seq.protection.TwoSampleScanSkellamPeakCaller.backgroundName

protected

WindowProcessor<CountScore> broad.pda.seq.protection.TwoSampleScanSkellamPeakCaller.backgroundProcessor

protected

Map<String, Map<Gene, Double> > broad.pda.seq.protection.TwoSampleScanSkellamPeakCaller.backgroundScanPvalues

protected

double broad.pda.seq.protection.TwoSampleScanSkellamPeakCaller.EXPRESSION\_PVALUE\_CUTOFF = 0.01

static

Transcriptome wide scan P value cutoff for expression of gene

Map<String, Collection<Gene> > broad.pda.seq.protection.TwoSampleScanSkellamPeakCaller.genes

protected

Logger broad.pda.seq.protection.TwoSampleScanSkellamPeakCaller.logger = Logger.getLogger(TwoSampleScanSkellamPeakCaller.class.getName())

staticprotected

int broad.pda.seq.protection.TwoSampleScanSkellamPeakCaller.MIN\_WINDOW\_COUNT = 10

staticprotected

double broad.pda.seq.protection.TwoSampleScanSkellamPeakCaller.pValueCutoffScan

protected

double broad.pda.seq.protection.TwoSampleScanSkellamPeakCaller.pValueCutoffSkellam

protected

TranscriptomeSpaceAlignmentModel broad.pda.seq.protection.TwoSampleScanSkellamPeakCaller.signalData

protected

String broad.pda.seq.protection.TwoSampleScanSkellamPeakCaller.signalName

protected

WindowProcessor<ScanStatisticScore> broad.pda.seq.protection.TwoSampleScanSkellamPeakCaller.signalProcessor

protected

int broad.pda.seq.protection.TwoSampleScanSkellamPeakCaller.stepSize

protected

TranscriptomeSpace broad.pda.seq.protection.TwoSampleScanSkellamPeakCaller.transcriptomeSpace

protected

double broad.pda.seq.protection.TwoSampleScanSkellamPeakCaller.trimPeakByQuantile

protected

int broad.pda.seq.protection.TwoSampleScanSkellamPeakCaller.windowSize

protected

---

The documentation for this class was generated from the following file:

- java/broad/pda/seq/protection/TwoSampleScanSkellamPeakCaller.java


---

Generated on Fri Aug 15 2014 15:11:03 for ESAT by  

 1.8.7
